# Nutrition and movement to improve quality of life in patients with knee osteoarthritis: the NUMOQUA study protocol for a randomised controlled trial

**DOI:** 10.1186/s13063-024-08048-2

**Published:** 2024-04-09

**Authors:** Elisabeth Höld, Sabine Chmelar, Tatjana Aubram, Gabriele Leitner, Stefan Nehrer, Oliver Neubauer, Karl-Heinz Wagner, Barbara Wondrasch

**Affiliations:** 1 Department of Health Sciences, St. Pölten University of Applied Sciences, St. Pölten, Austria; 2 Vienna Doctoral School of Pharmaceutical, Nutritional and Sport Science (PhaNuSpo), University of Vienna, Vienna, Austria; 3 Institute for Innovation Systems, St. Pölten University of Applied Sciences, St. Pölten, Austria; 4 Faculty of Health and Medicine, University for Continuing Education Krems, Krems, Austria; 5 Research Platform Active Ageing, University of Vienna, Vienna, Austria; 6 Department of Nutritional Sciences, University of Vienna, Vienna, Austria

**Keywords:** Osteoarthritis, GLA:D®, Exercise therapy, Nutrition therapy, Austrian Osteoarthritis Cuisine, Randomised controlled trial, Austria, Quality of Life

## Abstract

**Background:**

Osteoarthritis (OA) has long been considered as a degenerative disease of cartilage tissue resulting from bodily wear and tear. However, there is accumulating evidence that inflammation plays a key role in the pathogenesis of OA. In knee OA, the most common form of OA, exercise therapy as an effective component of early treatment addresses functional deficits, pain and inflammation. Since inflammation is critical for the development and progress of OA, anti-inflammatory therapies must be combined strategically.

In the course of the NUMOQUA project, an anti-inflammatory therapeutic diet named ‘Austrian Osteoarthritis Cuisine’ was developed. It is based on the framework of the New Nordic Diet combined with the food-based dietary guidelines of Austria, the guidelines for OA, the Austrian food culture and the principles of a sustainable diet. The present study examines the implementation of the ‘Austrian OA Cuisine’ combined with the evidence-based training programme GLA:D® (Good Life with osteoArthritis in Denmark) in Austrian patients with knee OA and the effects on quality of life, nutritional and inflammatory status, as well as oxidative stress parameters.

**Methods:**

A total of 60 participants aged 50 to 75 with knee OA will be included and randomly assigned either to the intervention group or the control group. All participants will undergo the GLA:D® programme in the first 6 weeks. Additionally, the intervention group will receive nutritional group training and individual nutritional counselling on the ‘Austrian OA Cuisine’ over 9 months. The control group will receive general information about a healthy lifestyle. Measurements at baseline and at 4 follow-up dates include nutritional, inflammatory and oxidative stress markers. Furthermore, anthropometric, behavioural and clinical data will be obtained. The recruitment process lasted from autumn 2022 to January 2024, followed by the intervention until October 2024.

**Discussion:**

The prevalence of OA is expected to increase in the future due to ongoing demographic changes and rising obesity rates. The expected results will provide important evidence on whether this interdisciplinary therapeutic approach could be a new, cost-effective and sustainable strategy to address the disease process of OA without negative side effects.

**Trial registration:**

ClinicalTrials.gov NCT05955300. Date of registration: 23rd of October 2023.

## Administrative information

Note: the numbers in curly brackets in this protocol refer to SPIRIT checklist item numbers. The order of the items has been modified to group similar items (see http://www.equator-network.org/reporting-guidelines/spirit-2013-statement-defining-standard-protocol-items-for-clinical-trials/).

**Table:** 

Title {1}	SPIRIT guidance: Descriptive title identifying the study design, population, interventions, and, if applicable, trial acronym. Nutrition and movement to improve quality of life in patients with mild to moderate knee osteoarthritis: the NUMOQUA study protocol for a randomised controlled trial
Trial registration {2a and 2b}.	SPIRIT guidance: Trial identifier and registry name. If not yet registered, name of intended registry. Item 2b is met if the register used for registration collects all items from the World Health Organization Trial Registration Data Set. ClinicalTrials.gov Identifier: NCT05955300
Protocol version {3}	SPIRIT guidance: Date and version identifier. 31/05/2023, Version 1
Funding {4}	SPIRIT guidance: Sources and types of financial, material, and other support. This investigator-initiated study is publicly funded by the Gesellschaft für Forschungsförderung Niederösterreich m.b.H, Austria (Life Science Call, 2020, LSC20-17)
Author details {5a}	SPIRIT guidance: Affiliations of protocol contributors. ^1^Department of Health Sciences, St. Pölten University of Applied Sciences, St. Pölten, Austria ^2^Vienna Doctoral School of Pharmaceutical, Nutritional and Sport Science (PhaNuSpo), University of Vienna, Vienna, Austria ^3^Institute for Innovation Systems, St. Pölten University of Applied Sciences, St. Pölten, Austria ^4^Faculty of Health and Medicine, University for Continuing Education Krems, Krems, Austria ^5^Research Platform Active Ageing, University of Vienna, Vienna, Austria ^6^Department of Nutritional Sciences, University of Vienna, Vienna, Austria
Name and contact information for the trial sponsor {5b}	SPIRIT guidance: Name and contact information for the trial sponsor. Gesellschaft für Forschungsförderung Niederösterreich m.b.H, Hypogasse 1, 3100 St. Pölten, Austria; telephone: + 43 2742 275 70–0; email: office@gff-noe.at (formerly NÖ Forschungs- und Bildungsges.m.b.H)
Role of sponsor {5c}	SPIRIT guidance: Role of study sponsor and funders, if any, in study design; collection, management, analysis, and interpretation of data; writing of the report; and the decision to submit the report for publication, including whether they will have ultimate authority over any of these activities. The funding body (Gesellschaft für Forschungsförderung NÖ m.b.H.) is neither involved in nor has any influence on any study-related aspect of this study such as study design, management, analysis, interpretation and dissemination of study results.

## Introduction

### Background and rationale {6a}

Osteoarthritis (OA) is the most prevalent arthritic disease worldwide and is characterised by pain, effusion and stiffness leading to functional decline and loss of quality of life (QL) [1]. Furthermore, OA is the 11th highest global cause of disability resulting in significant morbidity and costs to the healthcare system [1, 2]. Developing lifestyle-based strategies to counteract clinical symptoms associated with OA is a high priority for biomedical research.

OA has long been considered as a degenerative disease of cartilage resulting from bodily wear and tear that affects the entire joint structure including the fibrillation and degradation of articular cartilage, thickening of the subchondral bone, formation of osteophytes and hypertrophy of the joint capsule. In addition to these structural pathologic changes in the joint, there is accumulating evidence that inflammation plays a key role in the pathogenesis of OA [3]. Triggered by tissue damage and metabolic dysfunction, inflammatory processes within the joint (local inflammation) may contribute to the development of chronic low-grade inflammation (systemic inflammation) [3, 4]. Factors that contribute to chronic low-grade inflammation include excessive energy intake, nutrient deficiency, obesity and abnormal metabolites [5]. Chronic low-grade inflammation, in turn, promotes local inflammation, which increases pain and effusion in the joint [4]. Together, local and chronic low-grade inflammation correlate with clinical symptoms of OA, accelerate the progression of the disease and might even influence its onset [4, 5].

Knee OA is the most common form of OA. Its prevalence rises with age and peaks at around 50 years of age, affecting more than 250 million people worldwide. The global prevalence of knee OA in 2010 was estimated to be 3.8% and it was higher in females than in males [6]. Between 1990 and 2019, the global prevalence of knee OA increased by 122% [7]. The medical costs caused by OA amount to approximately 1–2.5% of the national gross domestic product, while indirect costs associated with, e.g. work loss or premature retirement involves further financial burden [8]. Due to ongoing demographic changes, the prevalence of knee OA is expected to increase further in the future, which emphasises the magnitude of this disorder and stresses the need for a paradigm shift towards early and effective treatment [6]. For example, a recent study expects a 38% increase of OA in Austria from 2019 to 2080 [9].

Physical activity and exercise therapy represent important and effective components of early treatment and are therefore highly recommended as a first-line treatment of OA [10]. Despite strong evidence from more than 50 randomised controlled trials (RCTs) in knee OA and 10 RCTs in hip OA supporting the efficacy of exercise therapy, education and weight loss, the implementation of these modalities is still insufficient and suboptimal in clinical practice [11]. This may be due to the fact that there is a need for a comprehensive patient- and context-specific approach to successfully implement the clinical guideline [12]. Good Life with osteoArthritis in Denmark (GLA:D®) represents such an evidence-based treatment plan for knee and hip OA consisting of patient education and exercise therapy [13]. Studies have shown that GLA:D® is feasible and effective in reducing pain and improving function and QL, at least in the short term [12]. The GLA:D® training programme is offered and carried out exclusively by certified GLA:D® physiotherapists. Those instructors use GLA:D® in their usual care of OA patients in Austria.

While current exercise therapy programmes address pain and physical as well as functional deficits of OA patients, there is a need for more effective therapeutic strategies targeting chronic low-grade inflammatory processes [4]. The combination of exercise therapy and diet represents a promising approach to address this issue: firstly evidence shows that exercise provokes anti-inflammatory effects directly by inhibition of the inflammatory pathway and the production of inflammatory factors [14] and also indirectly by improving body composition through reducing abdominal adiposity [15]. Secondly, the diet composition exerts an important influence on inflammatory processes and plays a critical role in inflammation-related diseases [16, 17]. The combination of these two inflammation-modulating therapies might not only counteract the progression of OA but may possibly even prevent the onset of OA and improve function and QL in the long term.

A Western diet, characterised on the one hand by a high intake of fat, cholesterol, protein, sugar, salt, processed and ‘fast foods’ [18] - especially meat products - and, on the other hand, by a low amount of plant-based foods including fresh fruit and vegetables, legumes, seeds and nuts [19], evokes a state of chronic low-grade inflammation [16]. Therefore, lifestyle changes that positively influence inflammatory processes should be part of ‘first-line’ interventions in the management of OA, thereby reducing the rate of the disease’s progression, reducing weight and improving health by minimising a patient’s risk or manifestation of other lifestyle-related conditions [20].

Further, a plant-based therapeutic diet that elicits beneficial effects on the patients’ inflammatory and oxidative stress status helps to reduce pain and to improve the QL. It also offers the possibility to reduce non-steroidal, anti-inflammatory and analgesic medications [21]. This means that a therapeutic diet, which is a major part of adequate and comprehensive nutrition therapy, should focus on foods with high content of inflammation-modulating ingredients, like polyphenols and vitamins in fruit or vegetables, high-quality fatty acids in vegetable oils (rich in alpha-linolenic acid) and fish (rich in long-chain polyunsaturated n-3 fatty acids) [19, 21, 22] and a high intake of fibre [21]. Furthermore, probiotics [23] and fasting [24] can improve inflammation-associated symptoms too.

However, patients face several barriers while implementing a therapeutic diet [25] as part of nutrition therapy. Besides a ‘lack of willpower’ and limitations in time and money, dietary habits and taste preferences are mainly responsible for the adherence to a therapeutic diet [26, 27]. Therefore, recommendations to implement an anti-inflammatory therapeutic diet such as the Mediterranean diet [28, 29] in Austria might not be attractive for patients as it does not correspond with regional needs and habits [30]. Furthermore, it can be observed that dietary recommendations focus mainly on health aspects and less on environmental issues [31], although food (production) has a major environmental impact [32] which should be considered in prevention as well as in therapy [33]. These factors still remain unconsidered in the treatment of patients with OA. As a consequence, there is a need for a tasty and sustainable therapeutic diet for patients with OA.

For tackling these limitations, the New Nordic Diet (NND), which was developed in the early 2000s by experts in the fields of nutrition, gastronomy, environmental issues, food culture and sensory science, focuses on the principles of health, gastronomic potential and sustainability. The fundamental guidelines are as follows: first, more calories from plant-based foods and less from meat; secondly, more foods from the sea and the lakes; and third, more foods from the wild countryside. These principles and guidelines can be applied to any region [34] including Austria. Furthermore, the NND focuses on specific regional products, which is also a characteristic of the Austrian cuisine. Especially vegetables and fruits (e.g. roots, cabbage, legumes, apples, pears, or berries), cereals (e.g. whole grain rye, oats, or barley), lake fish and rapeseed oil [35] correspond highly with Austrian needs [36]. Finally, the SYSDIET study showed that the NND has anti-inflammatory characteristics [37, 38], which make the NND the perfect basis for the OA therapy.

To our knowledge, no study has examined the influence of NND on the symptoms, QL and progression of OA. Nor does a local adaptation of the NND exist in Austria. Implementing the NND as the tasty and sustainable therapeutic diet Austrian OA Cuisine would have several advantages over other dietary recommendations for patients with OA: stronger adherence to the therapeutic diet by taking regional preferences into account [39] and therefore

- a better outcome in terms of a changed nutrition and inflammation status, a reduction of symptoms and increased joint function resulting in improved quality of life as well as
- a reduced environmental impact through the high amount of plant-based products, locally grown seasonal food and the high amount of organic products [40, 41]

With this research, we address the lack of knowledge concerning

- the impact of exercise therapy AND nutrition therapy on the symptoms, QL and progression of knee OA as well as
- the acceptance of the Austrian OA Cuisine in patients with knee OA as well as perceived social and cultural barriers [39] and supportive factors for the implementation.

### Objectives {7}

The main objectives of this study are, on the one hand, to analyse the effect of the nutrition therapy Austrian OA Cuisine combined with the GLA:D® training programme on QL in patients with mild to moderate knee OA. On the other hand, the study investigates the impact of a nutrition therapy combined with the GLA:D® training programme on symptoms, nutrition and inflammation status and oxidative stress markers, as well as joint function in patients with mild to moderate knee OA.

In particular, our main hypotheses are

- H1: Austrian OA Cuisine combined with the GLA:D® training programme will improve QL in patients with mild to moderate knee OA.
- H2: Austrian OA Cuisine combined with the GLA:D® training programme will reduce symptoms in patients with mild to moderate knee OA.
- H3: Austrian OA Cuisine combined with the GLA:D® training programme will improve the nutrition status in patients with mild to moderate knee OA.
- H4: Austrian OA Cuisine combined with the GLA:D® training programme will improve the inflammation status in patients with mild to moderate knee OA.
- H5: Austrian OA Cuisine combined with the GLA:D® training programme will improve the oxidative stress markers in patients with mild to moderate knee OA.

### Trial design {8}

This study is a parallel-group, two-arm superiority RCT to evaluate the impact of the nutrition therapy Austrian OA Cuisine combined with an evidence-based training programme (GLA:D®) in patients with mild to moderate knee OA in Lower Austria. The treatment combination of the nutrition therapy Austrian OA Cuisine and the GLA:D® training programme is compared with the treatment involving the GLA:D® training programme only.

Participants are informed about all aspects of the study and asked to sign a consent form. Participants are randomised at a 1:1 ratio (see {16a}). Staggered recruitment will be carried out to expand the recruitment phase and to handle all participants’ appointments and trainings more easily.

The duration of the intervention is 9 months. Within this intervention period, the intervention group undergoes the GLA:D® training programme for the first 6 weeks and receives the Austrian OA Cuisine for 9 months. The control group undergoes the GLA:D® training programme for 6 weeks and receives general information regarding a healthy lifestyle. During the 3 months follow-up phase, the intervention group members are expected to implement the Austrian OA Cuisine as well as the exercises of the GLA:D® training programme on their own into their daily life.

## Methods: participants, interventions and outcomes

### Study setting {9}

The study will be implemented in Lower Austria, which is a federal state in north-eastern Austria. Lower Austria is the country’s largest federal state with the second highest number residents (January 2023: 1,718,529 residents). Only Vienna, which is the capital city of Austria, has more residents than Lower Austria [42].

With an arthritis (knee, hip, finger) prevalence of 14.8% of all inhabitants aged at least 15 years, Lower Austria was in fourth place concerning this disease in Austria in 2019 [43].

The trainings and medical examinations are carried out at the David Institute in Krems (located in the centre of Lower Austria and rather close to the capital city Vienna). The David Institute is a regional competence centre for medical training and exercise medicine. Biochemical analyses are carried out at the University of Vienna and the University for Continuing Education Krems. Besides the David Institute, participants can also take part in the trainings at the St. Pölten University of Applied Sciences (also located in the centre of Lower Austria).

### Eligibility criteria {10}

The participants must meet the following inclusion criteria:

- Aged ≥ 50 to < 75 years
- A diagnosis of tibiofemoral OA with severity equivalent to Kellgren and Lawrence (K/L) grades 1 to 3

A potential participant is excluded if she/he reports suffering from diabetes mellitus, uncontrolled hypertension, acute/instable neurological or psychiatric diseases (e.g. depression), systematic inflammatory diseases (e.g. rheumatoid arthritis) or alcohol/drug abuse. Further exclusion criteria are obesity classes 2 and 3 (Body Mass Index [BMI] ≥ 35 kg/m^2^) [44] or if participants follow self-reported vegetarian or vegan eating habits.

During the informed-consent process, the prospective participants’ ability to understand the intervention and their German language skills are verified.

Regarding medication and supplementation, the following additional exclusion criteria have been defined:

- History of a cortisol therapy in the last 6 weeks prior to the study
- Therapy with autologous conditioned plasma in the last 6 weeks prior to the study
- Injection of hyaluronic acid in the knee in the last 12 weeks prior to the study
- Chronic anti-inflammatory medication (e.g. non-steroidal anti-inflammatory drugs)
- Intake of high-dose nutritional supplements (minimum wash-out of 6 weeks before study start)

The GLA:D® training programme is provided by experienced physiotherapists, who are educated as GLA:D® instructors.

Dietetic group training and individual nutritional counselling are delivered by experienced, registered dietitians of the St. Pölten University of Applied Sciences. The 24-h dietary recalls are administered by a nutritionist or dietitian.

### Who will take informed consent? {26a}

Persons interested in participating can register using an online registration form, by sending an email, or via telephone and the responsible dietitian will contact them.

During the initial appointment, which is conducted online via MS Teams or face to face at the St. Pölten University of Applied Sciences, the dietitian explains the study (background, protocol, different roles, importance of randomisation and adherence) to potential participants and reviews the consent form. Furthermore, the dietitian answers any questions that arise. When the study and its requirements are understood completely and the inclusion and exclusion criteria are met, the informed consent form is signed by the participant and the study coordinator. The participant and the study team each receive a signed version of the informed consent form. All eligible participants must complete the consent procedure before enrolment and randomisation.

### Additional consent provisions for collection and use of participant data and biological specimens {26b}

Independent of this study, the GLA:D® training programme demands the collection of health-related data from all participants for quality control purposes. Therefore, all participants sign the additional consent form of the GLA:D® training programme.

### Interventions

#### Explanation for the choice of comparators {6b}

The research question was based on the PICO (Patient, Intervention, Comparison, Outcome) principle and reads as follows: The effects of the GLA:D® training programme combined with the nutrition therapy Austrian OA Cuisine - compared to the GLA:D® training programme alone - on quality of life in patients with knee osteoarthritis. To answer this research question, a randomised controlled clinical trial is conducted.

Both the intervention group and the control group participate in the GLA:D® training programme, which has already been shown to reduce pain and improve function as well as QL [15]. To examine the synergistic effects, the intervention group receives, in addition to the GLA:D® programme, training and counselling concerning the implementation of the developed nutrition therapy named Austrian OA Cuisine (see {11a}). The control group receives general information about a healthy lifestyle.

#### Intervention description {11a}

The nutrition therapy Austrian OA Cuisine is based on the framework of the New Nordic Diet [34] combined with the food-based dietary guidelines of Austria [45], the guidelines for OA [22, 46], the typical Austrian cuisine (based on standard works of Austrian cookbooks [47, 48], initiatives to promote traditional and regional foods [49–52] and the principles of a sustainable diet [31].

The nutrition therapy consists of dietetic group training and individual nutritional counselling and is realised by an experienced dietitian. There are 4 group trainings covering 2 h each during the first 6 weeks of the intervention at the David Institute in Krems and at the University of Applied Sciences St. Pölten. The curriculum contains:

- General information concerning OA and its inflammatory characteristics
- The role of nutrition in the development and progression of OA [53]
- The principles of the Austrian OA Cuisine (e.g. largely plant-based, rich in n-3 fatty acids)
- The practical implementation in daily life (including a recipe booklet)
- A tasting of different dishes and practical exercises to increase food literacy (e.g. adapting own recipes) [54]
- Strategies for self-management, self-motivation, self-control and goal-setting [55]

The concept of the Austrian OA Cuisine provides adequate intake of energy and nutrients for the age group over 51 years [56], which is the age group most affected by OA. Besides, it takes the sustainability aspect into account - e.g. by giving preference to regional and seasonal products. The developed diet meets the recommendations for an anti-inflammatory diet [57].

After the end of group training, a comprehensive individual counselling will be provided once a month until 9 months after the study start, either face to face, by telephone, or virtually (MS Teams). The individual counselling consolidates the knowledge and competences and also monitors and motivates participants to keep implementing the therapeutic diet.

After 9 months of nutrition therapy, a follow-up of 3 months will be implemented. During this time, participants should continue to implement the Austrian OA Cuisine on their own.

The GLA:D® training programme consists of two patient education sessions (approx. 1.5 h per session) and two supervised, group-based exercise therapy sessions (approx. 1 h per session) per week over the first 6 weeks of the intervention. The education sessions include information about OA, its symptoms, treatment options and self-management strategies. The group training is based on the NEMEX programme [58] and includes exercises to strengthen hip and knee muscles as well as neuromuscular exercises to improve joint control and stability. Although it is a standardised programme, the exercises can be individualised and adapted to the patients’ needs [13, 59]. The education and training sessions take place at the David Institute Krems and the St. Pölten University of Applied Sciences.

### Criteria for discontinuing or modifying allocated interventions {11b}

Participants can revoke their willingness to participate at any time, even without giving reasons, and withdraw from the clinical study without any disadvantages for their further medical care.

The principal investigator may terminate a participation in the study prematurely without obtaining the participants' consent beforehand. Reasons for this include:

- Participants do not longer meet the inclusion criteria of the clinical study.
- The treating physician comes to the conclusion that continued participation in the clinical study is not in the participants' best interest.
- The study coordinator decides to terminate the entire clinical study, or to terminate only one subject’s participation prematurely.

Furthermore, participants must attend at least three out of four dietetic group training sessions and eight out of twelve GLA:D® training programme sessions. If a participant cannot meet this quota, he/she is excluded from the study.

### Strategies to improve adherence to interventions {11c}

At the first appointment prior to the start of the study, the potential participants receive a detailed description of the study (background, randomisation, intervention, results, etc.) to be sure that they have understood everything correctly before giving their consent to participate in the study (see ‘Who will take informed consent? {26a}’). This initial appointment is conducted by the dietitian who has the most contact with the participants during the study. This supports personal contact and adequate relationships from the beginning.

Throughout the study, the central study staff members - mainly the dietitian - maintain close contact to further strengthen human relationships. The participants are reminded about all appointments via text message 1 week beforehand. The data collection at the different measurement times is well organised with time slots to reduce waiting times for the participants. During the initial 6-week group training (GLA:D® training programme and dietetic group training), the participants build intensive relationships with each other and with the central study staff members due to the intensity of the programme. Through the monthly performed 24-h dietary recalls (see {12}) and the subsequent individual nutritional counselling, the participants in the intervention group are encouraged to continue implementing the recommendations of the Austrian OA Cuisine after the end of the group phase [60].

Throughout the course of the study, participants receive selected interim results (for example, the body composition measurement) to promote motivation. After the study is finished, they will also be provided their data from the biochemical measurements, if they wish so.

Participants receive a financial compensation and a goodie bag with highly relevant food groups of the Austrian OA Cuisine - mainly organic and locally produced vegetable oils and nuts. Additionally, participants take part in the evidence-based GLA:D® training programme guided by experienced GLA:D® physiotherapists free of charge. Regularly, the costs of physiotherapy are not fully covered by insurance in Austria. As a central component of the intervention, the intervention group members participate in holistic nutrition therapy counselled by an experienced dietitian, also without additional costs. Besides, the control group has the possibility to discuss their eating habits and biochemical data with a dietitian after the study is finished.

### Relevant concomitant care permitted or prohibited during the trial {11d}

Participants are instructed not to take high-dose dietary supplements during the study (e.g. vitamin supplements that exceed the reference values [56] for the nutrient intake). Any changes in medication or supplementation are queried at the various data collection points.

#### Provisions for post-trial care {30}

Study participation entails only minimal risk because nutrition therapy [29, 61] - in this case, the Austrian OA Cuisine - and the GLA:D® training programme [13] are associated with extremely low health risks. Therefore, no special provisions for post-study care are included.

### Outcomes {12}

Data are collected five times over a period of 12 months: at baseline (T0), 6 weeks after the start of the intervention (T1; T0 + 6 weeks), 3 months after the start of the intervention (T2; T0 + 3 months), 9 months after the start of the intervention (T3; T0 + 9 months) and after the follow-up period (T4; T0 + 12 months), including the assessment of blood-based biochemical, anthropometric and behavioural parameters at four data collection points (T0, T2, T3 and T4). The collection of demographic and anamnestic data and the execution of the functional tests take place at all five data collection points.

The primary outcome is the quality of life subscale (QL) of the Knee Injury and Osteoarthritis Outcome Score (KOOS) [62]. The KOOS is used to assess the joint function and patients’ opinion about their knee and associated problems and is a valid and reliable tool compared to others in several studies [62]. The KOOS score for knee-related quality of life (QL) is one of five subscales of the KOOS questionnaire. The QL score is calculated based on four 5-point Likert scale questions, in which each question is assigned a score between 0 and 4. According to the standardised score calculation (www.koos.nu), the mean of the observed items is divided by 4, multiplied by 100 and finally subtracted from 100. A QL score of 100 indicates no symptoms, while a score of 0 means extreme symptoms. The KOOS is valid and reliable and was compared to other instruments in several studies [62].

To evaluate the symptoms of the knee OA and associated problems, the following parameters are analysed:

- Further KOOS subscales collect the participants' opinion about their knee pain (Pain), knee function in daily living (ADL), knee function in sport and recreation (Sport/Rec) and other knee-associated symptoms (Symptoms). The latter is relevant for testing H2. Equally to the KOOS subscale QL, each subscore is calculated based on 5-point Likert scale questions, where each question is assigned a score from 0 to 4. Following the standard procedure for the subscore calculation, the mean of the subscore questions is divided by 4, multiplied by 100 and finally subtracted from 100. A subscore of 100 indicates no symptoms, while a score of 0 points to extreme symptoms.
- To evaluate the functional status of the participants' knee, the 40-m fast-paced walk test (40 MFPWT) and the 30-s chair stand test (30 SCST) are performed. The 40 MFPWT is a valid and reliable tool to measure gait speed and to monitor changes in knee OA patients’ physical function over time [63]. The 30 SCST is a valid and reliable tool to measure lower limb strength in patients with knee OA [64].

The following nutritional parameters are used as the basis for testing the third hypothesis:

- Anthropometrical parameters:
  - Body height [cm], body weight [kg] and body fat [%] as measured using Seca mBCA 555. The device measures body weight with a scale, body height with ultrasound length measurement and body composition by the voltage drop of the alternating current in one step
  - Waist circumference [cm] to calculate the waist-to-height ratio as measured using an ergonomic, stepless and extendible measuring tape

- Biochemical parameters:
  - Lipide profile (plasma α-linolenic acid, plasma linoleic acid, plasma total polyunsaturated fatty acids, plasma Ω6:Ω3 ratio), measured as described in [65];
  - Carotenoids (α- and β-carotene, lutein, lycopene), measured as described in [66, 67] and;
  - Vitamins D [68] and K, measured as described [66].

- Behavioural parameters:
  - Dietary behaviour is measured using an adapted version of the Vienna Food Record (VFR), which is a paper-based prospective food record and can be completed without an interview or oral instruction. The VFR consists of 182 food items which meet the requirements of Austrian adults [69]. For this study, adaptations to assess fat quality (fat fish, vegetable oils, nuts and seeds) more precisely were implemented. To support participants in answering the VRF, an additional booklet with real portion size pictures corresponding to the food items are provided to the participants.

Additionally, the oxidative stress and inflammatory status are determined as secondary outcomes based on the following biochemical parameters:

- Oxidative stress parameters (ferric reducing ability potential, malondialdehyde, oxidised and reduced glutathione, damage to desoxyribonucleic acid), measured as previously described [70].
- Inflammation biomarkers (C-reactive protein, interleukins (IL)-1β, IL-6, IL-18, tumour necrosis factor alpha, matrix metalloproteinase (MMP)-3), measured by multiplex assays as described previously [71].

In addition to the outcome parameters, the following demographic and anamnestic data are collected:

- Sex, gender, employment status, residence, number of people in the household, income, immigration background [72]
- Consumption of alcohol and smoking behaviour
- Sleep quality
- Clinical information (menopausal status, regular intake of supplements and drugs, medical history, joint complaints, sick leave)
- Actual self-perceived health status and pain
- Physical activity level at work, leisure time activities
- The general physical activity level is assessed using the ‘International Physical Activity Questionnaire Short Form’ (IPAQ-SF) [73]: The IPAQ-SF asks seven questions to assess ‘vigorous-intensity’ and ‘moderate-intensity’ physical activity as well as ‘walking’ and ‘sitting’. Participants indicate the time in minutes or hours for each activity level. Based on this information, three levels of physical activity (low, moderate, high) are calculated and expressed in the metabolic equivalent of task (MET) minutes per week.
- Feedback on the participants’ satisfaction with the GLA:D® training programme will be collected at T1 and T4 by using a visual analogue scale.

Besides these measurement appointments, participants take part in monthly 24-h dietary recalls via telephone, except for the first, which is conducted in person at the second measurement point (T1). Interviews are performed by trained project staff in a structured manner based on the framework of the multiple-pass method [74–76]. Except for one 24-h dietary recall, which gathers data on weekend eating behaviour, the other seven 24-h dietary recalls collect data on eating behaviour from Monday to Thursday. To reduce bias, participants know only the calendar week for the 24-h dietary recalls but not the exact day. All 24-h dietary recalls are analysed using the programme nut.s nutritional software (https://www.nutritional-software.at) in its latest version (April 2022) [77].

Furthermore, qualitative data are collected 7 months after each intervention round has started. This will be done via three focus groups with intervention group participants. It will be conducted to analyse the acceptance of the Austrian OA Cuisine and identify perceived social and cultural barriers as well as supportive factors for its integration into daily life. By taking these data into account, a more holistic understanding of the success of the implementation of the nutrition therapy will be possible. Provisional topics used to guide the focus groups will be developed in advance. The focus groups will be managed by a dietitian of the study staff. The dietitian will be responsible for recording, documenting and creating a summary analysis.

#### Participant timeline {13}

**Fig. 1:**
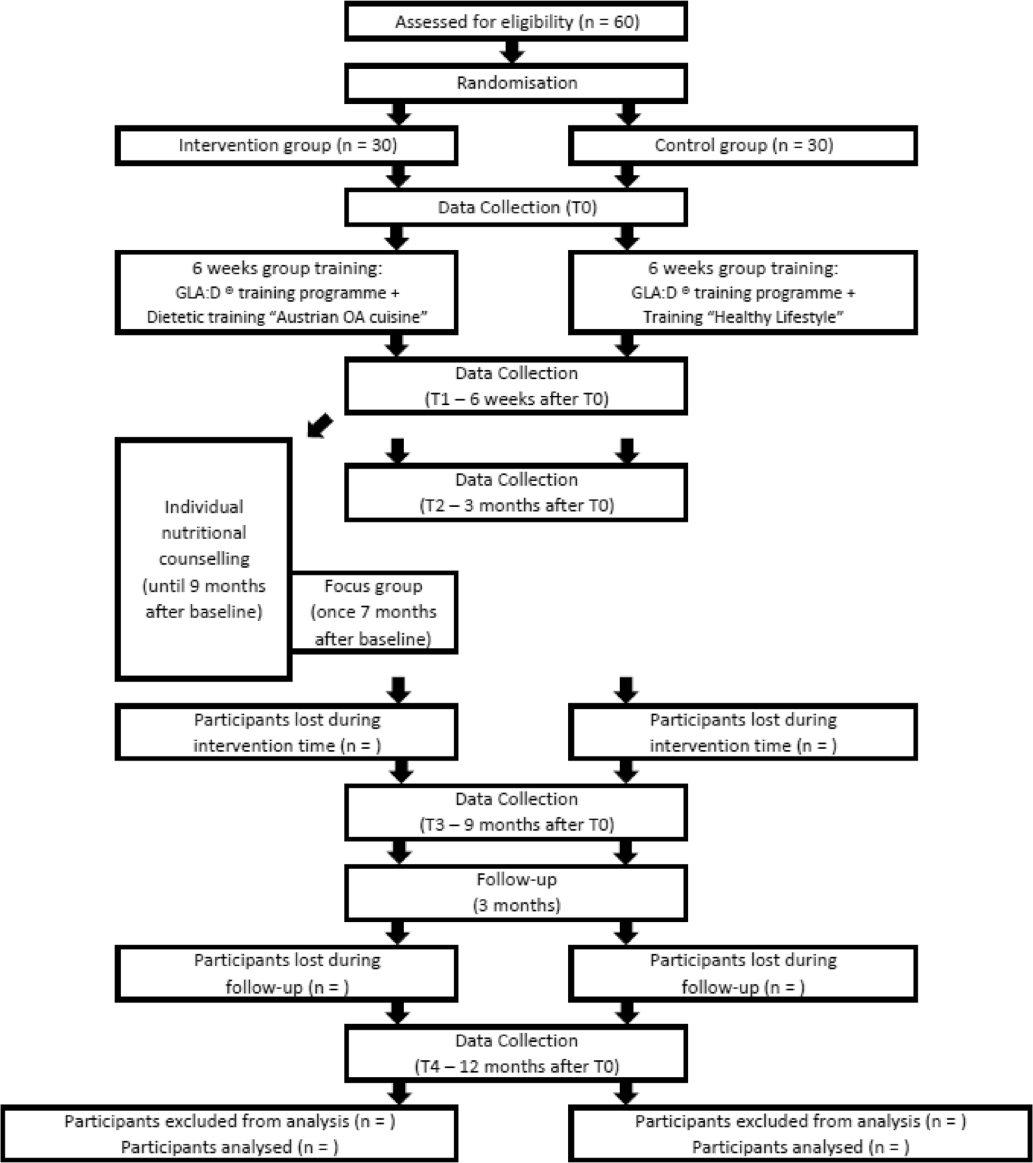
Flow diagram NUMOQUA

**Fig. 2:**
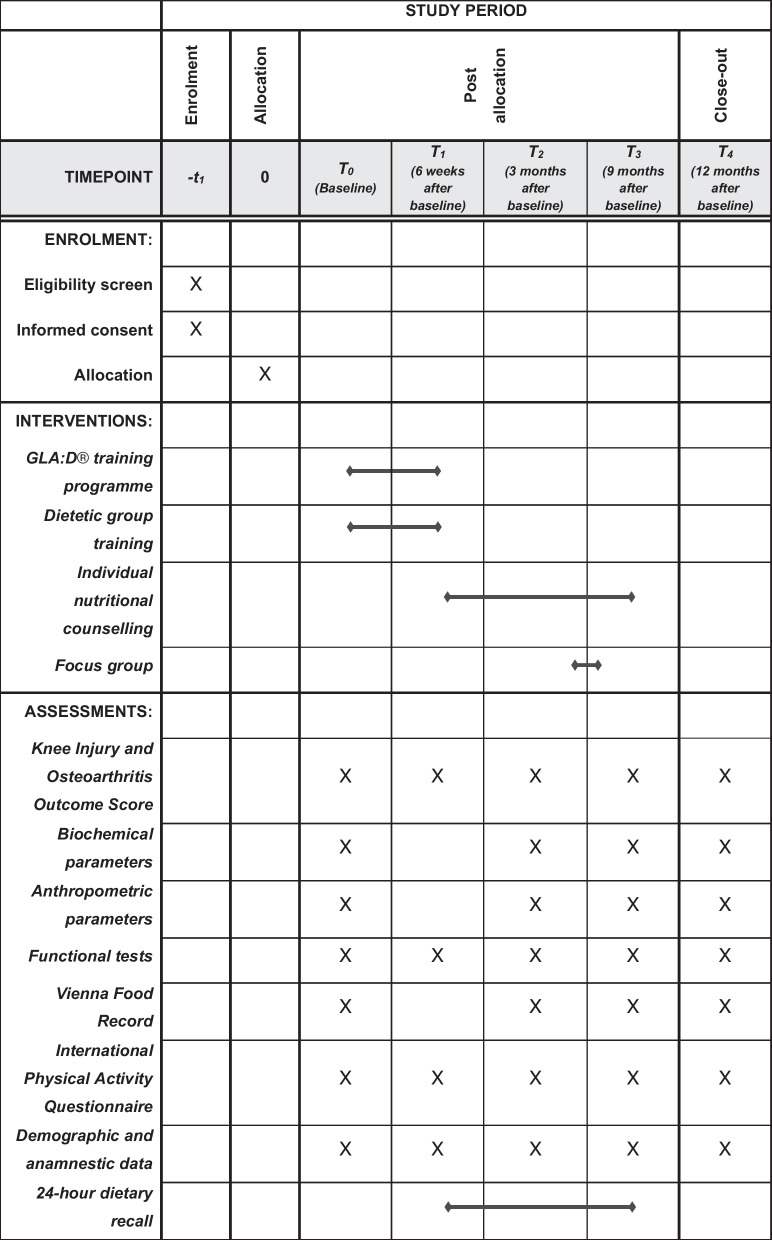
SPIRIT figure NUMOQUA

The procedure of the study is presented in a flow diagram (Fig. 1). The SPIRIT Figure for this study is provided in Fig. 2.

### Sample size {14}

An a priori power calculation was performed using G-Power [78] and establishing an ANOVA (F-test) between two measures and two groups for the primary endpoint, the quality of life subscale of the Knee Injury and Osteoarthritis Outcome Score, demonstrating that 50 participants (25 in each group) are required to reveal differences at the 5% significance level, with 90% power, using large effect size (1.1) as reported by previous studies [79, 80]. A change of 8–10 points is considered the minimally important change (MIC) and the standard deviation is set to 15 [81]. Considering a 20% drop-out ratio, we decided to include 30 participants per group (60 participants in total).

### Recruitment {15}

The St. Pölten University for Applied Sciences has carried out other studies regarding OA in the past, which means that there is a database of people interested in OA studies. In the first step, these are contacted by email and informed about this planned study. Press releases are placed in newspapers and magazines—especially in local ones—and information is posted on social media. Flyers are distributed at relevant congresses, at clinicians specialising in OA, and events at the St. Pölten University of Applied Sciences. Furthermore, networks like self-help groups (OA forum Austria), the Austrian Association of Dietitians and the intranet of the St. Pölten University of Applied Sciences are used to reach out to interested people.

## Assignment of interventions: allocation

### Sequence generation {16a}

After a successful recruitment of suitable participants, they are stratified according to sex, randomised using the *Random Allocation Software* [82] and allocated either to the intervention or to the control group.

### Concealment mechanism {16b}

Once a consenting number of suitable participants per intervention round (*n* = 20) have been recruited, subjects will be randomised into intervention group (IG) and control group (CG). This is done by allocating participants to a sequential ID number. Using the *Random Allocation Software* [82], these ID numbers are allocated either to the intervention group or to the control group. The Random Allocation Software will not release the randomisation code until the patient has been recruited into the trial.

### Implementation {16c}

The treatment allocation will be performed by one member of the study team. After the participants’ randomisation, group allocation will be communicated via email.

## Assignment of interventions: Blinding

### Who will be blinded {17a}

A blinding of study participants will not be possible because of the obvious differences between the intervention group and the control group. Blinding will be implemented for the medical staff and the outcome assessors of the measurements and the following analyses (see ‘Data management {19}’).

#### Procedure for unblinding if needed {17b}

It is an open-label study and therefore unblinding will not occur.

## Data collection and management

### Plans for assessment and collection of outcomes {18a}

During the registration process, contact data of all interested individuals are collected. The data are deleted if the person disagrees to participate in the study or does not meet the criteria.

All parameters as described in the subsection ‘Outcomes (see {12})’ are taken in a group testing setting with appropriate COVID-19 safety measures, e.g. testing, vaccination, hygiene procedures by the staff of the St. Pölten University of Applied Sciences either at the university itself or at David Institute Krems.

Due to the need for blood sampling and anthropometric measurements at T0, T2, T3 and T4, data collection is conducted in the morning in a fasting state. Anthropometric measurements are conducted with light clothing and carried out by dietitians and nutritionists.

The functional tests (40 MFPWT and 30 SCST) are also carried out with light and casual clothing as well as shoes after the anthropometric measurements. A fasting state is not necessary. Demographic and anamnestic data as well as quality-of-life parameters (KOOS and EQ-5D-5L) are collected via online questionnaires using LimeSurvey (https://www.limesurvey.org/de) as online survey tool. For the data entry, the participants use prepared laptops at the measurement site. Blood samples are collected by medical staff from the David Institute. Fresh blood samples are centrifuged and processed according to the specifications of each biochemical parameter (see ‘Outcomes {12}’) at the University of Continuing Education Krems (located in close proximity to the David Institute). All processed blood samples are stored at − 80 °C and analysed at the University of Continuing Education Krems and at the University of Vienna.

Besides this data collection at the measurement appointments, 24-h dietary recalls are conducted as described in Outcomes ({12}).

### Plans to promote participant retention and complete follow-up {18b}

As with the other measurement time points, participants receive a reminder for the follow-up appointment. After the last measurement, the financial compensation is paid and the participants receive their personal test results. In addition, the members of the control group then have the possibility to consult the dietitian.

### Data management {19}

The participants agree that for the purpose and in the course of this study, the St. Pölten University of Applied Sciences collects and processes behavioural, anthropometric, demographic and anamnestic data as well as data from the KOOS and functional tests (see ‘Outcomes {12}’). Furthermore, the participants agree that the University of Continuing Education Krems and the University of Vienna transmit the biochemical data (see ‘Outcomes {12}’) in pseudonymised form to the St. Pölten University of Applied Sciences.

The participants’ data are pseudonymised and assigned a fixed identification code. This code is stored safely in a password-protected file, accessible only by the study coordinator (BW) and central staff members (SC, TA, EH). Thus, it is only possible for persons who are entrusted with the evaluation of the data to infer the identity of the participants.

Data acquisition and data processing are performed using commercial software. All data obtained are stored in computer files in encrypted form at the server of the St. Pölten University of Applied Sciences. The handling of the data complies with the European General Data Protection Regulation, the Austrian Data Protection Act and the recommendations of the Austrian Ethics Committees.

Quantitative data are stored and analysed via the statistical software package SPSS at servers of the St. Pölten University of Applied Sciences. To be able to merge the data from the online questionnaires, anthropometric measurements and the blood sample data, a defined coding system is used. Therefore, central staff members (SC, TA) take part in every measurement and organise the participants and coding system. Hence, every participant gets their identification code for the anthropometric measurements and the blood samples and a personalised link including password for the online questionnaire via *Limesurvey*. The identification code ensures that the study staff do not know to which group each participant belongs. To support participants in completing the online questionnaires, prepared laptops and supporting staff members are available. The questionnaires are checked on site by the study staff in terms of completeness and correctness related to the specifications of the questionnaires. Study data from online questionnaires are exported and saved as a comma-separated values (CSV) file after every measurement.

Results of the biochemical analysis are uploaded by the University of Continuing Education Krems and the University of Vienna via encrypted Excel files to a protected research server at the St. Pölten University of Applied Sciences and imported into SPSS by the study coordinator.

Anthropometric data are collected on site using the identification codes in the encrypted and password-protected cloud application of SECA (https://www.seca.com/de_at/555.html). Also, these data are exported and saved as a comma-separated values (CSV) file after every measurement.

Furthermore, the St. Pölten University of Applied Sciences performs data back-ups on a daily basis. All signed informed consent forms as well as all collected pen-and-paper sheets are stored in a locked place, accessible only for the study coordinator and a central staff member (SC). All data are stored at the St. Pölten University of Applied Sciences for 10 years after the study’s end. After that, all data are to be destroyed.

To ensure data quality, the study coordinator controls the data at regular intervals, which includes a check of the range of values or double data entry. Results of all analyses are reported in an aggregated and strictly anonymous form.

### Confidentiality {27}

All collected data are kept confidential as described in ‘Data management’ (see {19}). Only the study coordinator (BW) and central staff members (SC, TA, EH) can open the password-protected file to link personal information and code. The final pseudonymised dataset in SPSS will be used by the study staff for analyses, publications and reports only for this study.

### Plans for collection, laboratory evaluation and storage of biological specimens for genetic or molecular analysis in this trial/future use {33}

Not applicable as no biological specimens for genetic or molecular analysis are collected.

## Statistical methods

### Statistical methods for primary and secondary outcomes {20a}

In this study, the focus is on the comparison of data collected at the beginning of the study (T0) and at the end of the study (T4; T0 + 12 months). For all statistical tests, the significance level is set at *p* < 0.05 in accordance with scientific standards. Data are analysed using IBM SPSS Statistics 28 or greater. Normal distribution of metric variables is tested with Shapiro–Wilk-Test and Q-Q-diagrams. For interpreting the results correctly, we also conduct a descriptive analysis showing the arithmetic mean and standard deviation of metric outcome parameters for each group. To control for outliers, we also report the minimum and maximum for each group in addition to a supportive graphical analysis. For interval and nominal scaled variables, we conduct a frequency analysis. To compare the effects of an evidence-based training programme (GLA:D®) with and without the nutrition therapy Austrian OA Cuisine, we conduct an analysis of variance (ANOVA) based on a mixed-linear model of repeated measures looking for assessing effects within each group (e.g. between baseline and the other data collection points) and between groups. In case variables are non-normal or non-metric in scale, we use the Friedman Test, respectively [83]. The group variable (intervention group or control group) serves as the independent variable and the observed outcome parameters (see {12}) serve as the dependent variable.

#### Interim analyses {21b}

There will be no planned interim analysis or stopping guidelines for medical reasons besides the exclusion criteria (see ‘Eligibility criteria {10}’) because no potentially harmful outcomes are expected based on the GLA:D® training programme or the nutrition therapy (see ‘Provisions for post-trial care {30}’).

### Methods for additional analyses (e.g. subgroup analyses) {20b}

Depending on the data and data distribution, subgroup analyses are carried out, e.g. data is clustered according to the body weight. In addition, different correlations - like between the clinical data and the dietary behaviour of the participants - can be analysed.

### Methods in analysis to handle protocol non-adherence and any statistical methods to handle missing data {20c}

Nonadherence data will be analysed by the intention to treat (ITT) method [84]. For handling missing data, multiple imputation will be performed [85].

### Plans to give access to the full protocol, participant-level data and statistical code {31c}

It is not planned to give third parties access, neither to the full protocol, nor the participant data, nor the statistical code. Only the data of the GLA:D® training programme will possibly be used for transnational research in the framework of the GLA:D® International Network in the long term. Therefore, participants sign an additional consent form as described in ‘Additional consent provisions for collection and use of participant data and biological specimens {26b}’.

## Oversight and monitoring

### Composition of the coordinating centre and trial steering committee {5d}

The complexity of OA treatment demands interdisciplinary teams and innovative approaches. The trial steering committee meets these requirements and combines the life sciences of physiotherapy, nutritional sciences, dietetics, human medicine and biomedicine in a unique consortium. Based on their expertise, the study staff members take responsibility for the defined work packages: project management, dissemination, recruitment, physiotherapy, nutritional sciences and dietetics, data collection respectively data processing and analysis.

The trial steering committee meets twice a year. The central study staff members of the St. Pölten University of Applied Sciences meet bi-weekly. The persons responsible for the individual work packages meet as required during the course of the study. The study coordinator has the final responsibility.

### Composition of the data monitoring committee, its role and reporting structure {21a}

A Data Monitoring Committee (DMC) has been established (BW, EH, TA, SC). The DMC ensures correct implementation of the data collection and secure data storage. The collected data are reviewed after each measurement point.

The committee takes all necessary measures for data encryption and for protection of the participants’ data against unauthorised access. If security problems are identified, the Data Monitoring Committee informs the study members and also the funding agency if needed and initiates all necessary steps. In addition, the data protection officer of the study centre can be consulted by the DMC.

### Adverse event reporting and harms {22}

Although the risk of occurring complaints and adverse side effects in this study is considered to be very low, systematic management of side effects is implemented. Participants are advised to report complaints during exercise therapy directly to the physiotherapist, who then forwards this information to the study coordinator. Every potential adverse event is documented in detail and collected at the study centre.

The study coordinator informs the Ethics Committee and the study’s insurance company if necessary.

### Frequency and plans for auditing trial conduct {23}

The whole consortium meets at least twice a year, depending on the project’s tasks.

The study coordinator (BW) and central staff members (SC, EH, TA) meet twice a month to review the general study conduct. Furthermore, the staff members responsible for the GLA:D® training programme (BW, MF, AH, TA) as well as the staff members responsible for the Austrian OA Cuisine and nutritional counselling (SC, EH, GL) meet twice a month. Moreover, central nutrition-related decisions are supervised by KHW and ON. The Data Monitoring Committee (DMC) meets as described in the subsection ‘Composition of the data monitoring committee, its role and reporting structure {21a}’.

The study coordinator (BW) reports to the Ethics Committee as well as to the funding organisation at least once a year. If adaptations of the study protocol should become necessary, further notifications are made to these organisations by the study coordinator.

### Plans for communicating important protocol amendments to relevant parties (e.g. trial participants, ethical committees) {25}

Approval for protocol modification is sought from the Ethics Committee of Vienna as well as the funding organisation (Gesellschaft für Forschungsförderung Niederösterreich m.b.H, Austria) by using the appropriate documents. Upon approval of all changes, the study coordinator (BW) updates the clinical study registry. If protocol modifications differ significantly from what was explained to participants when signing the informed consent, the participants are immediately informed about all changes and re-consent is sought by a study staff member (SC).

### Dissemination plans {31a}

The consortium has agreed on authorship guidelines [86], scientific and general dissemination activities in relevant scientific and clinical fields.

All dissemination activities will be documented and reported by the responsible consortium member. When it comes to scientific dissemination, the study protocol and the study results will be published in peer-reviewed open-access journals as well as in the trial register ClinicalTrials.gov (Identifier: NCT05955300). The study and its results will be presented at scientific conferences on a national and international level. Participants will receive a lay summary of the study results. Beyond that various activities are planned to disseminate to the general population, e.g. at special events like the ‘European Researchers’ Night’, study website posts, press releases, or radio and TV contributions.

## Discussion

The number of people affected by OA will continue to increase, mainly based on the well-known demographic changes [6, 9] and the Western lifestyle characterised by unfavourable eating habits and limited physical activity leading to a rising prevalence of overweight and obesity [87]. This situation will have far-reaching consequences for those affected and for society as a whole. On the one hand, OA has a direct negative impact on the quality of life of those affected due to the typical pain, stiffness and decline of functionality [1]. On the other hand, society is also confronted with serious consequences like loss of workforce, early retirement and increasing costs for the healthcare system [8].

The conventional therapy of OA with non-steroidal anti-inflammatory drugs has significant side effects, e.g. gastrointestinal complications, renal disturbances and cardiovascular events [88], and is cost-intensive as well. Additional nutritional therapy has played a lesser role in the guidelines on the treatment of OA, so far, and is largely limited to reducing any excess weight that may be present [55, 89, 90]. The theory behind this is to reduce the load on the knee joint and thereby reduce cartilage degradation. However, recent data imply an additional inflammatory process which plays a vital role in the pathogenesis of OA [3].

This situation highlights the need for a novel and interdisciplinary approach: The NUMOQUA study implements a unique lifestyle-based therapy by combining the GLA:D® training programme and the Austrian OA Cuisine - a diet that beneficially modulates inflammation and oxidative stress. The NUMOQUA study aims to increase quality of life and reduce pain as well as low-grade inflammation without serious health risks and with lower costs [12, 91, 92].

Additionally, the NUMOQUA approach provides the following benefits which go far beyond OA:

- Consistent pain relief makes movement easier and could lead to a higher long-term physical activity level. This creates the opportunity to influence other physical parameters, e.g. body weight [93] or non-communicable diseases which are linked to nutrition and physical activity such as diabetes type 2, cardiovascular diseases or some types of cancer [91, 94–96].
- This is also confirmed by the World Health Organization, which claims that behavioural factors like an unhealthy diet and a lack of physical activity are among the main reasons for chronic diseases and the associated healthcare costs [97].
- The Austrian OA Cuisine can positively influence other inflammation-related diseases, e.g. atopic dermatitis [98] or inflammatory bowel disease [99].
- The analysed acceptance of the Austrian OA Cuisine can help to understand barriers and thresholds against a mainly plant-based nutrition style. By understanding these processes, better strategies for implementing a largely plant-based diet in other population groups can be developed. This is of great importance for health and environment, as the EAT-Lancet report stated in 2019 [32].
- The Austrian OA cuisine promotes local food producers and cycles and contributes to the United Nations Sustainable Development Goal 12 (Responsible consumption and production) [100].

In summary, OA is a complex chronic disease of public interest which requires an interdisciplinary therapy approach. The NUMOQUA study aims to increase quality of life and reduce negative health consequences by combining of the GLA:D® training programme with the largely plant-based anti-inflammatory and anti-oxidant nutrition therapy named Austrian OA Cuisine. The results will provide scientific evidence for the efficacy of a cost-effective, innovative approach to address this relevant disease.

## Trial status

This is the protocol version 1 from 31/05/2023. Recruitment started in October 2022 and has ended in January 2024.

## Data Availability

Only the data monitoring committee (see ‘Composition of the data monitoring committee, its role and reporting structure {21a}’) will have access to the final trial dataset.

## Abbreviations

ADL: Activities of daily living
AH: Andrea Haas
ANOVA: Analysis of variance
BMI: Body mass index
BW: Barbara Wondrasch
CG: Control group
CRP: C-reactive protein
CSV: Comma-separated values
DMC: Data Monitoring Committee
EH: Elisabeth Höld
EQ-5D-5L: European Quality of Life 5 Dimensions 3 Level Version
EUR: Euro
GL: Gabriele Leitner
GLA:D®: Good Life with osteoArthritis in Denmark
ID: Identifier
IG: Intervention group
IL: Interleukin
IPAQ-SF: International Physical Activity Questionnaire Short Form
KHW: Karl-Heinz Wagner
KOOS: Knee Injury and Osteoarthritis Outcome Score
KP: Kerstin Prock
OA: Osteoarthritis
MET: Metabolic equivalent of task
MF: Magdalena Führer
MIC: Minimally important change
MMP: Matrix metalloproteinase
MS: Microsoft
NEMEX: Neuromuscular exercise
NND: New Nordic Diet
NUMOQUA: Nutrition and movement to improve quality of life with knee osteoarthritis
n-3: Omega 3
ON: Oliver Neubauer
QL: Quality of life
RCT: Randomised controlled trial
SC: Sabine Chmelar
SDG: Sustainable Development Goals
SMS: Short message service
SN: Stefan Nehrer
SPSS: Statistical Package for the Social Sciences
TA: Tatjana Aubram
TNF: Tumour necrosis factor
UAS: University of Applied Sciences
VFR: Vienna Food Record
30 SCST: 30-S chair stand test
40 MFPWT: 40-M fast-paced walk test

## References

[1] Bruyère O, Honvo G, Veronese N, Arden NK, Branco J, Curtis EM (2019). An updated algorithm recommendation for the management of knee osteoarthritis from the European Society for Clinical and Economic Aspects of Osteoporosis, Osteoarthritis and Musculoskeletal Diseases (ESCEO). Semin Arthritis Rheum.

[2] Tan BY, Ding BTK, Pereira MJ, Skou ST, Thumboo J, Car J (2020). Collaborative model of care between Orthopaedics and allied healthcare professionals trial (CONNACT) – a feasibility study in patients with knee osteoarthritis using a mixed method approach. BMC Musculoskelet Disord.

[3] Berenbaum F (2013). Osteoarthritis as an inflammatory disease (osteoarthritis is not osteoarthrosis!). Osteoarthritis Cartilage.

[4] Robinson WH, Lepus CM, Wang Q, Raghu H, Mao R, Lindstrom TM (2016). Low-grade inflammation as a key mediator of the pathogenesis of osteoarthritis. Nat Rev Rheumatol.

[5] Wang X, Hunter D, Xu J, Ding C (2015). Metabolic triggered inflammation in osteoarthritis. Osteoarthr Cartil.

[6] Davis AM, Kennedy D, Wong R, Robarts S, Skou ST, McGlasson R (2018). Cross-cultural adaptation and implementation of Good Life with osteoarthritis in Denmark (GLA:DTM): group education and exercise for hip and knee osteoarthritis is feasible in Canada. Osteoarthr Cartil.

[7] Long H, Liu Q, Yin H, Wang K, Diao N, Zhang Y (2022). Prevalence trends of site-specific osteoarthritis from 1990 to 2019: findings from the global burden of disease study 2019. Arthritis Rheumatol.

[8] Hunter DJ, Schofield D, Callander E (2014). The individual and socioeconomic impact of osteoarthritis. Nat Rev Rheumatol.

[9] Hitzl W, Stamm T, Kloppenburg M, Ritter M, Gaisberger M, van der Zee-Neuen A (2022). Projected number of osteoarthritis patients in Austria for the next decades - quantifying the necessity of treatment and prevention strategies in Europe. BMC Musculoskelet Disord.

[10] Wellsandt E, Golightly Y (2018). Exercise in the management of knee and hip osteoarthritis. Curr Opin Rheumatol.

[11] Gay C, Chabaud A, Guilley E, Coudeyre E (2016). Educating patients about the benefits of physical activity and exercise for their hip and knee osteoarthritis. Systematic literature review. Ann Phys Rehabil Med..

[12] Skou ST, Bricca A, Roos EM (2018). The impact of physical activity level on the short- and long-term pain relief from supervised exercise therapy and education: a study of 12,796 Danish patients with knee osteoarthritis. Osteoarthr Cartil.

[13] Skou ST, Roos EM (2017). Good Life with osteoArthritis in Denmark (GLA:DTM): evidence-based education and supervised neuromuscular exercise delivered by certified physiotherapists nationwide. BMC Musculoskelet Disord.

[14] Deng X, Xu H, Hao X, Liu J, Shang X, Xu T (2023). Effect of moderate exercise on osteoarthritis. EFORT Open Rev.

[15] Pedersen BK (2019). The physiology of optimizing health with a focus on exercise as medicine. Annu Rev Physiol.

[16] Christ A, Lauterbach M, Latz E (2019). Western diet and the immune system: an inflammatory connection. Immunity.

[17] Minihane AM, Vinoy S, Russell WR, Baka A, Roche HM, Tuohy KM (2015). Low-grade inflammation, diet composition and health: current research evidence and its translation. Br J Nutr.

[18] Manzel A, Muller DN, Hafler DA, Erdman SE, Linker RA, Kleinewietfeld M (2014). Role of ‘Western diet’ in inflammatory autoimmune diseases. Curr Allergy Asthma Rep.

[19] Cena H, Calder PC (2020). Defining a healthy diet: evidence for the role of contemporary dietary patterns in health and disease. Nutrients..

[20] Dean E, Gormsen HR (2012). Prescribing optimal nutrition and physical activity as ‘first-line’ interventions for best practice management of chronic low-grade inflammation associated with osteoarthritis: evidence synthesis. Arthritis.

[21] Bärebring L, Winkvist A, Gjertsson I, Lindqvist H (2018). Poor dietary quality is associated with increased inflammation in Swedish patients with rheumatoid arthritis. Nutrients.

[22] Messina OD, Vidal Wilman M, Vidal Neira LF (2019). Nutrition, osteoarthritis and cartilage metabolism. Aging Clin Exp Res.

[23] Vaghef-Mehrabany E, Alipour B, Homayouni-Rad A, Sharif SK, Asghari-Jafarabadi M, Zavvari S (2014). Probiotic supplementation improves inflammatory status in patients with rheumatoid arthritis. Nutrition.

[24] Müller HF, de Toledo FW, Resch KL (2001). Fasting followed by vegetarian diet in patients with rheumatoid arthritis: a systematic review. Scand J Rheumatol.

[25] Pellegrini CA, Ledford G, Chang RW, Cameron KA (2018). Understanding barriers and facilitators to healthy eating and physical activity from patients either before and after knee arthroplasty. Disabil Rehabil.

[26] de Mestral C, Khalatbari-Soltani S, Stringhini S, Marques-Vidal P (2020). Perceived barriers to healthy eating and adherence to dietary guidelines: nationwide study. Clin Nutr.

[27] Pinho MGM, Mackenbach JD, Charreire H, Oppert JM, Bárdos H, Glonti K (2018). Exploring the relationship between perceived barriers to healthy eating and dietary behaviours in European adults. Eur J Nutr.

[28] Bortoluzzi A, Furini F, Scirè CA (2018). Osteoarthritis and its management - epidemiology, nutritional aspects and environmental factors. Autoimmun Rev.

[29] Veronese N, Stubbs B, Noale M, Solmi M, Luchini C, Smith TO (2017). Adherence to a Mediterranean diet is associated with lower prevalence of osteoarthritis: data from the osteoarthritis initiative. Clin Nutr.

[30] Mithril C, Dragsted LO, Meyer C, Tetens I, Biltoft-Jensen A, Astrup A (2013). Dietary composition and nutrient content of the New Nordic diet. Public Health Nutr.

[31] von Koerber K, Waldenmaier J, Cartsburg M (2020). Nutrition and the guiding principle of sustainability. Global challenges and problem-solving approaches on a national and international UN level. Ernahrungs Umschau..

[32] Willett W, Rockström J, Loken B, Springmann M, Lang T, Vermeulen S (2019). Food in the Anthropocene: the EAT–Lancet commission on healthy diets from sustainable food systems. The Lancet.

[33] Müller O, Jahn A, Gabrysch S (2018). Planetary health: Ein umfassendes Gesundheitskonzept. Dtsch Arztbl.

[34] Mithril C, Dragsted LO, Meyer C, Blauert E, Holt MK, Astrup A (2012). Guidelines for the New Nordic diet. Public Health Nutr.

[35] The KM, Diet N (2017). Towards the North by inspiration from the South. Ernaehrungs Umschau Int.

[36] Kammer für Arbeiter und Angestellte für Steiermark: Ernährungstrends unter der Lupe. Was gibt’s Neues rund ums Thema Essen? https://stmk.arbeiterkammer.at/service/broschuerenundratgeber/konsument/20180207_Ernaehrungstrends-unter-der-Lupe-barrf.pdf (2017). Accessed 10 Oct 2023.

[37] Tuomainen M, Kärkkäinen O, Leppänen J, Auriola S, Lehtonen M, Savolainen MJ (2019). Quantitative assessment of betainized compounds and associations with dietary and metabolic biomarkers in the randomized study of the healthy Nordic diet (SYSDIET). Am J Clin Nutr.

[38] Roager HM, Vogt JK, Kristensen M, Hansen LBS, Ibrügger S, Mærkedahl RB (2019). Whole grain-rich diet reduces body weight and systemic low-grade inflammation without inducing major changes of the gut microbiome: a randomised cross-over trial. Gut.

[39] Micheelsen A, Holm L, O’Doherty JK (2013). Consumer acceptance of the New Nordic diet an exploratory study. Appetite.

[40] Meltzer HM, Brantsæter AL, Trolle E, Eneroth H, Fogelholm M, Ydersbond TA (2019). Environmental sustainability perspectives of the Nordic diet. Nutrients.

[41] Saxe H (2014). The New Nordidietc diet is an effective tool in environmental protection: it reduces the associated socioeconomic cost of diets. Am J Clin Nutr.

[42] STATISTIK AUSTRIA: Bevölkerung zu Jahres-/Quartalsanfang. https://www.statistik.at/statistiken/bevoelkerung-und-soziales/bevoelkerung/bevoelkerungsstand/bevoelkerung-zu-jahres-/-quartalsanfang (2023). Accessed 9 Mar 2023.

[43] Klimont J. Österreichische Gesundheitsbefragung 2019. Hauptergebnisse des Austrian Health Interview Surve (ATHIS) und methodische Dokumentation. Bundesministerium für Soziales, Gesundheit, Pflege und Konsumentenschutz, Statistik Austria. 2020. https://www.statistik.at/fileadmin/publications/Oesterreichische-Gesundheitsbefragung2019_Hauptergebnisse.pdf. Accessed 10 Oct 2023.

[44] World Health Organization. Obesity: preventing and managing the global epidemic: report of a WHO consultation. (WHO technical report series: 894) WHO Library Cataloguing-in-Publication Data. 2000. https://iris.who.int/handle/10665/42330?locale-attribute=en&show=full. Accessed 3 Mar 2022. 11234459

[45] Bundesministerium für Soziales, Gesundheit, Pflege und Konsumentenschutz. Die österreichische Ernährungspyramide. 2020. https://broschuerenservice.sozialministerium.at/Home/Download?publicationId=617. Accessed 26 Mar 2023.

[46] Bannuru RR, Osani MC, Vaysbrot EE, Arden NK, Bennell K, Bierma-Zeinstra SMA (2019). OARSI guidelines for the non-surgical management of knee, hip, and polyarticular osteoarthritis. Osteoarthritis Cartilage.

[47] Plachutta E (2020). Die Plachutta Kochschule.

[48] Plachutta E, Wagner C (1993). Die gute Küche: das österreichische Jahrhundert Kochbuch.

[49] Agrarmarkt Austria Marketing GesmbH: Genussregionen Niederösterreich. https://www.genussregionen.at/en/region/lower-austria (2023). Accessed 13 Apr 2023.

[50] Bundesministerium für Land- und Forstwirtschaft, Regionen und Wasserwirtschaft (BML): Das isst Österreich! https://www.das-isst-oesterreich.at/ (2023). Accessed 13 Apr 2023.

[51] Bundesministerium für Land- und Forstwirtschaft, Regionen und Wasserwirtschaft (BML):: Traditionelle Lebensmittel in Österreich. https://info.bml.gv.at/themen/lebensmittel/trad-lebensmittel.html (2023). Accessed 13 Apr 2023.

[52] NÖ Dorf- und Stadterneuerung GmbH DORN: So schmeckt Niederösterreich. https://www.soschmecktnoe.at/ (2023). Accessed 13 Apr 2023.

[53] Thomas S, Browne H, Mobasheri A, Rayman MP (2018). What is the evidence for a role for diet and nutrition in osteoarthritis?. Rheumatology.

[54] Vidgen HA, Gallegos D (2014). Defining food literacy and its components. Appetite.

[55] Kolasinski SL, Neogi T, Hochberg MC, Oatis C, Guyatt G, Block J (2020). 2019 American College of rheumatology/arthritis foundation guideline for the management of osteoarthritis of the hand, hip, and knee. Arthritis Care Res.

[56] Deutsche Gesellschaft für Ernährung, Österreichische Gesellschaft für Ernährung, Schweizerische Gesellschaft für Ernährungsforschung, Schweizerische Vereinigung für Ernährung. Referenzwerte für die Nährstoffzufuhr. 2nd ed, 7th updated ed. Neustadt an der Weinstraße: Neuer Umschau Buchverlag GmbH; 2021.

[57] Adam O (2017). Ernährungsmedizinische Aspekte in der Rheumatologie. Phys Med Rehab Kuror.

[58] Ageberg E, Link A, Roos EM (2010). Feasibility of neuromuscular training in patients with severe hip or knee OA: the individualized goal-based NEMEX-TJR training program. BMC Musculoskelet Disord.

[59] Roos EM, Barton CJ, Davis AM, McGlasson R, Kemp JL, Crossley KM (2018). GLA: D to have a high-value option for patients with knee and hip arthritis across four continents: good life with osteoArthritis from Denmark. Br J Sports Med.

[60] Greaves CJ, Sheppard KE, Abraham C, Hardeman W, Roden M, Evans PH (2011). Systematic review of reviews of intervention components associated with increased effectiveness in dietary and physical activity interventions. BMC Public Health.

[61] Pitaraki EE (2017). The role of Mediterranean diet and its components on the progress of osteoarthritis. J Frailty Sarcopenia Falls.

[62] Samuel AJ, Kanimozhi D (2019). Outcome measures used in patient with knee osteoarthritis: with special importance on functional outcome measures. Int J Health Sc.

[63] Motyl JM, Driban JB, McAdams E, Price LL, McAlindon TE (2013). Test-retest reliability and sensitivity of the 20-meter walk test among patients with knee osteoarthritis. BMC Musculoskelet Disord.

[64] Kroman SL, Roos EM, Bennell KL, Hinman RS, Dobson F (2014). Measurement properties of performance-based outcome measures to assess physical function in young and middle-aged people known to be at high risk of hip and/or knee osteoarthritis: a systematic review. Osteoarthritis Cartilage.

[65] Nemeth M, Millesi E, Wagner KH, Wallner B (2018). Saliva cortisol responses to altered plasma PUFA patterns in guinea pigs. Br J Nutr.

[66] Jakob E, Elmadfa I (1995). Rapid HPLC assay for the assessment of vitamin K1, A, E and beta-carotene status in children (7–19 years). Int J Vitam Nutr Res.

[67] Neubauer O, Reichhold S, Nics L, Hoelzl C, Valentini J, Stadlmayr B (2010). Antioxidant responses to an acute ultra-endurance exercise: impact on DNA stability and indications for an increased need for nutritive antioxidants in the early recovery phase. Br J Nutr.

[68] Beckman Coulter GmbH: Access 25(OH) Vitamin D Total - 25(OH) vitamin D. https://www.beckmancoulter.com/products/immunoassay/access-vitamin-d ( 2017). Accessed 10 Apr 2023.

[69] Putz P, Kogler B, Bersenkowitsch I (2019). Reliability and validity of assessing energy and nutrient intake with the Vienna food record: a cross-over randomised study. Nutr J.

[70] Grindel A, Guggenberger B, Eichberger L, Pöppelmeyer C, Gschaider M, Tosevska A (2016). Oxidative stress, DNA damage and DNA repair in female patients with diabetes mellitus type 2. Plos One.

[71] Kardos D, Marschall B, Simon M, Hornyák I, Hinsenkamp A, Kuten O (2019). Investigation of cytokine changes in Osteoarthritic knee joint tissues in response to hyperacute serum treatment. Cells.

[72] United Nations Economic Commission for Europe. Conference of European Statisticians Recommendations for the 2020 Censuses of Population and Housing. 2015. https://unece.org/fileadmin/DAM/stats/publications/2015/ECECES41_EN.pdf. Accessed 11 May 2021.

[73] Lee PH, Macfarlane DJ, Lam TH, Stewart SM (2011). Validity of the International Physical Activity Questionnaire Short Form (IPAQ-SF): a systematic review. Int J Behav Nutr Phys Act.

[74] MRC Epidemiology Unit, University of Cambridge School of Clinical Medicine: Measurement Toolkit - 24-hour dietary recalls. https://beta.measurement-toolkit.org/diet/subjective-methods/24-hour-dietary-recall#ref17 (2023). Accessed 27 Mar 2023.

[75] Blanton CA, Moshfegh AJ, Baer DJ, Kretsch MJ (2006). The USDA automated multiple-pass method accurately estimates group total energy and nutrient intake 1,2. J Nutr.

[76] Moshfegh AJ, Rhodes DG, Baer DJ, Murayi T, Clemens JC, Rumpler WV (2008). The US Department of Agriculture automated multiple-pass method reduces bias in the collection of energy intakes. Am J Clin Nutr.

[77] dato Denkwerkzeuge: nut.s science. https://www.nutritional-software.at/content/nuts-software/nuts-science/ Accessed 22 Mar 2023.

[78] Heinrich-Heine-Universität Düsseldorf: G*Power. https://www.psychologie.hhu.de/arbeitsgruppen/allgemeine-psychologie-und-arbeitspsychologie/gpower (2023). Accessed 5 May 2023.

[79] Edwards PK, Ackland TR, Ebert JR (2013). Accelerated weightbearing rehabilitation after matrix-induced autologous chondrocyte implantation in the tibiofemoral joint: early clinical and radiological outcomes. Am J Sports Med.

[80] Hernández-Guillén D (2022). Talus mobilization-based manual therapy is effective for restoring range of motion and enhancing balance in older adults with limited ankle mobility: a randomized controlled trial. Gait Posture.

[81] Roos EM, Lohmander LS (2003). The Knee injury and Osteoarthritis Outcome Score (KOOS): from joint injury to osteoarthritis. Health Qual Life Outcomes.

[82] Saghaei M (2004). Random allocation software for parallel group randomized trials. BMC Med Res Methodol.

[83] Field A (2013). Discovering Statistics Using IBM SPSS Statistics.

[84] Dodd S, White IR, Williamson P (2012). Nonadherence to treatment protocol in published randomised controlled trials: a review. Trials.

[85] Beesley LJ, Bondarenko I, Elliot MR, Kurian AW, Katz SJ, Taylor JM (2021). Multiple imputation with missing data indicators. Stat Methods Med Res.

[86] Medical University of Vienna: Good Scientific Practice. Ethics in Science and Research. Guidelines of the Medical University of Vienna. https://www.meduniwien.ac.at/web/fileadmin/content/forschung/pdf/MedUni_Wien_GSP-Richtlinien_2017.pdf (2017). Accessed 10 May 2021.

[87] Chong B, Jayabaskaran J, Kong G, Chan YH, Chin YH, Goh R (2023). Trends and predictions of malnutrition and obesity in 204 countries and territories: an analysis of the Global Burden of Disease Study 2019. EClinicalMedicine.

[88] Harirforoosh S, Asghar W, Jamali F (2013). Adverse effects of nonsteroidal antiinflammatory drugs: an update of gastrointestinal, cardiovascular and renal complications. J Pharm Pharm Sci.

[89] Deutsche Gesellschaft für Orthopädie und Orthopädische Chirurgie. S2k-Leitlinie Gonarthrose. AWMF online. 2018. https://register.awmf.org/assets/guidelines/033-004l_S2k_Gonarthrose_2018-01_1-abgelaufen.pdf. Accessed 10 May 2021.

[90] Fernandes L, Hagen KB, Bijlsma JWJ, Andreassen O, Christensen P, Conaghan PG (2013). EULAR recommendations for the non-pharmacological core management of hip and knee osteoarthritis. Ann Rheum Dis.

[91] Wallace TC, Bailey RL, Blumberg JB, Burton-Freeman B, Chen CYO, Crowe-White KM (2020). Fruits, vegetables, and health: A comprehensive narrative, umbrella review of the science and recommendations for enhanced public policy to improve intake. Crit Rev Food Sci Nutr.

[92] Casas-Agustench P, Megías-Rangil I, Babio N, on behalf of the CODINUCAT Governing Board (2020). Economic benefit of dietetic-nutritional treatment in the multidisciplinary primary care team. Nutr Hosp.

[93] Mozaffarian D (2020). Dietary and policy priorities to reduce the global crises of obesity and diabetes. Nat Food.

[94] Craddock JC, Neale EP, Peoples GE, Probst YC (2019). Vegetarian-based dietary patterns and their relation with inflammatory and immune biomarkers: a systematic review and meta-analysis. Adv Nutr.

[95] Piepoli MF, Abreu A, Albus C, Ambrosetti M, Brotons C, Catapano AL (2020). Update on cardiovascular prevention in clinical practice: a position paper of the European Association of preventive cardiology of the European society of cardiology. Eur J Prev Cardiolog.

[96] Evert AB, Dennison M, Gardner CD, Garvey WT, Lau KHK, MacLeod J (2019). Nutrition therapy for adults with diabetes or prediabetes: a consensus report. Diabetes Care.

[97] World Health Organization Regional Office for Europe. European Food and Nutrition Action Plan 2015–2020. 2015. https://iris.who.int/bitstream/handle/10665/329405/9789289051231-eng.pdf?sequence=1.. Accessed 26 Mar 2023.

[98] Venter C, Meyer RW, Nwaru BI, Roduit C, Untersmayr E, Adel-Patient K (2019). EAACI position paper: Influence of dietary fatty acids on asthma, food allergy, and atopic dermatitis. Allergy.

[99] Wark G, Samocha-Bonet D, Ghaly S, Danta M (2021). The role of diet in the pathogenesis and management of inflammatory bowel disease: a review. Nutrients.

[100] United Nations: THE 17 GOALS. https://sdgs.un.org/goals (2023). Accessed 26 Mar 2023.

